# Beyond the amyloid hypothesis: leveraging human-centered complex *in vitro* models to decode Alzheimer’s disease etiology

**DOI:** 10.3389/ftox.2025.1753572

**Published:** 2026-01-09

**Authors:** Matthew Price, Francesca Pistollato

**Affiliations:** 1 School of Clinical and Experimental Sciences, University of Southampton, Southampton, United Kingdom; 2 Research and Toxicology, Humane World for Animals, Brussels, Belgium

**Keywords:** Alzheimer’s disease, complex *in vitro* models, environmental toxicants, gut-brain axis, infectious hypothesis, neuroinflammation, organoids, organ-on-a-chip

## Abstract

Alzheimer’s disease (AD) is a complex neurodegenerative condition and the leading cause of dementia worldwide. Treatments that safely and effectively counteract disease progression are currently lacking. While the formation of amyloid plaques has long been considered the leading hypothesis of disease onset, growing evidence suggests that the emergence of AD could be driven by a combination of underlying factors that promote chronic neuroinflammation, including pathogenic infections, environmental toxicants, and disruptions along the gut-brain axis. Traditional nonclinical models of AD, such as monolayer cell cultures and transgenic mice, struggle to capture the complexity of the disease as it occurs in humans. Human-centered complex *in vitro* models (CIVMs), including cerebral organoids and microfluidic organ-on-a-chip (OOC) technologies, provide greater physiological relevance by more closely recapitulating key cellular and molecular features of the human brain and disease mechanisms. In this mini review, we evaluate recent advances in CIVMs and how they are being leveraged to investigate emerging hypotheses of AD etiology. Cerebral organoids and OOC platforms can consistently replicate neuropathological hallmarks of neurodegeneration in response to pathogenic or environmental insults, including blood-brain barrier disruption, amyloid-β accumulation, tau hyperphosphorylation, and glial activation. We also highlight early efforts to model the gut–brain axis using organoid and multi-OOC systems, demonstrating how microbiota-derived factors can affect neural processes. Collectively, these studies show that human-centered CIVMs can be applied to both recreate and mechanistically disentangle interrelated pathological processes to an extent beyond that afforded by animal models, thus offering new opportunities to identify causal mechanisms and potential therapeutic targets.

## 1 Introduction

Alzheimer’s disease (AD) remains one of the most prevalent neurological disorders worldwide, accounting for 60%–70% of all dementia cases^1^. In 2021, an estimated 57 million people were living with dementia, with this figure being projected to nearly double every 20 years^2^.

Despite significant investment in basic and translational research, treatments that effectively and safely act on the evolution of the disease are currently lacking. Available drugs such as N-methyl-D-aspartate receptor antagonists (memantine) and cholinesterase inhibitors (donepezil, galantamine, rivastigmine) are largely symptomatic and do not suppress disease progression. Since 2021, the first anti-amyloid antibodies (aducanumab, lecanemab, donanemab) have been approved to target amyloid-β (Aβ) plaques, a pathological hallmark of AD^3^. These are considered disease-modifying therapies as they can slow cognitive decline in patients with mild cognitive impairment or mild dementia due to AD, but are associated with significant risks, including brain swelling and bleeding^4^.

The heterogeneity of patients and absence of reliable early diagnostic biomarkers hinder progress, as demonstrated by age-related shifts in Aβ positivity (Young and Mormino, 2022). Adding to the complexity, AD is a highly multifaceted condition, varying in age of onset, genetic risk factors, pathological processes and progression patterns, as well as the presence of comorbidities (Duara and Barker, 2022). This diversity makes it unlikely that treatments aimed at a single target will be effective across the entire patient population.

Over the past 15 years, new hypotheses beyond the amyloid cascade have emerged to explain the complex etiopathology of AD (Zhang et al., 2024). Several are interrelated, with dysfunctional metabolism, chronic inflammation, and environmental factors playing a critical role, along with ageing and genetics. These hypotheses include: (i) systemic inflammation caused by pathogen infections (Seaks and Wilcock, 2020; Bruno et al., 2023; Brown and Heneka, 2024), (ii) the impact of long-term exposure to environmental pollutants (Dhapola et al., 2024), and (iii) the role of microbiota and gut dysbiosis in the induction of neuroinflammation through the gut-brain axis (GBA) (Logan et al., 2023; Seo and Holtzman, 2024; Dhanawat et al., 2025).

Traditional nonclinical models of AD have relied on both *in vivo* and *in vitro* models that replicate key disease hallmarks. Transgenic mice that express genetic variants identified in familial AD (Zhong et al., 2024) can develop amyloid plaques, neurofibrillary tangles, gliosis, and mild cognitive deficits. Yet, even with amyloid accumulation, they frequently fail to show significant neuronal loss (Sanchez-Varo et al., 2022). Moreover, animal models do not replicate disease pathogenesis as it occurs in humans (Veening-Griffioen et al., 2019), failing to develop the comorbidities and risk factors commonly associated with sporadic, late-onset AD (Marshall et al., 2023). Even with optimized or humanized animal models, inherent interspecies differences in metabolism, gut microbiome, immune function, and epigenetic regulation considerably limit their external validity (Pound and Ritskes-Hoitinga, 2018). The inadequacy of traditional research models, coupled with their design being grounded in flawed or reductionist theories of disease etiology, has likely contributed to hindering a full understanding of disease complexity and played a relevant role in AD drug development failures.

In recent years, the use of human-centered complex *in vitro* models (CIVMs) such as cerebral organoids and organ-on-a-chip (OOC) systems, often derived from human induced pluripotent stem cells (hiPSCs) or primary cells, have deepened our understanding of AD pathology. CIVMs offer improved physiological relevance over traditional 2D monolayer cell cultures and animal models by better mimicking the complexities of the human brain and disease processes (Sreenivasamurthy et al., 2023; Dolciotti et al., 2025). Although 2D models have contributed significantly to AD research and remain widely used, they fall outside the scope of this review, which focuses on CIVMs that recapitulate multicellular and microenvironmental features more comprehensively. Furthermore, patient-derived CIVMs can be used to study individual differences in disease progression and response to treatment, possibly informing personalized medicine approaches (Lopes and Guil-Guerrero, 2025). These human-centered platforms can also be leveraged to explore novel etiological hypotheses underlying AD onset and to elucidate their potential interconnections.

**FIGURE 1:**
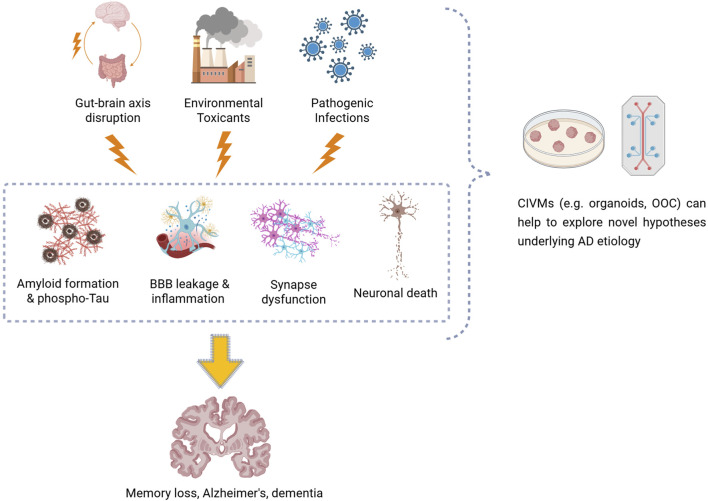
Diagram illustrating how gut–brain axis disruption, environmental toxicants, and pathogen infections could contribute to the development of Alzheimer’s disease–related neuropathology, such as amyloid plaque formation, neurofibrillary tau tangles, blood–brain barrier leakage, neuroinflammation, synaptic dysfunction, and neuronal loss. Complex *in vitro* models, including brain organoids and organ-on-a-chip systems, enable these mechanisms to be replicated and studied under controlled and human-relevant conditions.

In this mini review, we highlight recent applications of human-centered CIVMs (Figure 1), particularly cerebral organoids and single- or multi-OOC systems, and how they have been applied to investigate: (i) the potential impact of pathogen infections on neuroinflammation and AD; (ii) the effects of environmental pollutants on neurodegeneration and AD risk; and (iii) how alteration of gut microbiota and the GBA may drive neuroinflammation and neurodegeneration in AD. Together, these CIVM-based studies (summarized in Table 1) offer valuable human-relevant insights into AD pathogenesis, helping to uncover disease mechanisms and identify potential therapeutic targets.

**TABLE 1:** Summary of studies using complex *in vitro* models (CIVMs) to investigate (1) pathogenic, (2) environmental, and (3) gut-brain axis mechanisms relevant to Alzheimer’s disease.

CIVM	Operating principles	Cellular composition	Treatment	Key effects	Reference
(1) Pathogen-related factors					
Cerebral organoid	• Dynamic culture (orbital shaker) • Periodic medium refreshment • Hydrogel-embedded (Matrigel)	• hiPSC-derived neurons, astrocytes, microglia	HIV-1	• Microglial infection and pro-inflammatory activation	Boreland et al. (2024)
Cerebral organoid	• Static culture • Periodic medium refreshment • Hydrogel-embedded (PEGDMA)	• Primary human neurons, astrocytes, microglia	HIV-1	• Neuronal loss and synapse dysregulation • Astrogliosis • Microglial infection and pro-inflammatory activation	Dos Reis et al. (2020)
Cerebral organoid	• Dynamic culture (orbital shaker) • Periodic medium refreshment • Hydrogel-embedded (Matrigel)	• hiPSC-derived neurons, astrocytes, microglia, NPCs	HIV-1	• Neuronal upregulation of apoptotic markers and downregulation of neurotransmitter transporters • Microglial infection and pro-inflammatory activation	Kong et al. (2024)
Cerebral organoid	• Dynamic culture (orbital shaker) • Periodic medium refreshment • Hydrogel-embedded (Matrigel)	• hESC-derived neurons, astrocytes • hiPSC-derived microglia	HIV-1	• Microglial infection and pro-inflammatory activation • Inflammatory activation of neurons, astrocytes, and neural stem cells	Martinez-Meza et al. (2025)
Cerebral organoid	• Dynamic culture (orbital shaker) • Periodic medium refreshment • Self-aggregating	• hiPSC-derived neurons, astrocytes, microglia	HIV-1	• Microglial infection and pro-inflammatory activation	Narasipura et al. (2025)
Cortical organoid	• Dynamic culture (orbital shaker) • Periodic medium refreshment • Self-aggregating	• hiPSC-derived neurons, NPCs, radial glia	HSV-1	• Aβ accumulation mainly occurs in bystander cells, and not in HSV-1-infected cells	Abrahamson et al. (2021)
Cortical brain tissue model (3D)	• Static culture • Periodic medium refreshment • Biomaterial-based scaffold (porous silk protein sponges)	• hNSC-derived neurons, astrocytes	HSV-1	• Aβ (1–42) fibril-like formation • Neuronal APP, BACE1 downregulation and PSEN1/2 upregulation • Astrogliosis and pro-inflammatory activation	Cairns et al. (2020)
Brain–like tissue model (3D)	• Static culture • Periodic medium refreshment • Biomaterial-based scaffold (porous silk protein sponges)	• hNSC-derived neurons, astrocytes	HSV-1	• Aβ plaque formation and accumulation • Phosphorylated tau accumulation • Astrogliosis and pro-inflammatory activation • Reduced extracellular glutamate release	Cairns et al. (2025)
Neuronal cell culture (3D)	• Static culture • Periodic medium refreshment • Hydrogel-embedded (Matrigel)	• hNSC-derived neurons	HSV-1	• Aβ fibril accumulation and co-localization with HSV-1 and human herpesvirus 6A/B	Eimer et al. (2018)
Forebrain organoid	• Static culture • Periodic medium refreshment • Self-aggregating	• hiPSC-derived neurons, astrocytes, oligodendrocyte progenitors, NPCs	HSV-1	• Reduced neurite length • Elevated tau hyperphosphorylation, oligomerization, and production of 4R-tau	Ijezie et al. (2024)
Cerebral organoid	• Dynamic culture (orbital shaker) • Periodic medium refreshment • Hydrogel-embedded (Matrigel)	• hiPSC-derived neurons and microglia	HSV-1	• Aβ plaque formation	Oh et al. (2025)
Cerebral organoid	• Dynamic culture (orbital shaker) • Periodic medium refreshment • Hydrogel-embedded (Matrigel)	• hiPSC-derived neurons, astrocytes, microglia	HSV-1	• Microglia/astrocyte gliosis and pro-inflammatory activation	Qiao et al. (2020)
Cerebral organoid	• Dynamic culture (orbital shaker) • Periodic medium refreshment • Hydrogel-embedded (Matrigel)	• hESC-derived neurons, astrocytes, microglia	HSV-1	• Aβ deposition • Neuron loss • Gliosis • Microglial pro-inflammatory activation	Qiao et al. (2022)
Cerebral organoid	• Dynamic culture (orbital shaker) • Periodic medium refreshment • Hydrogel-embedded (Matrigel)	• hiPSC-derived neurons, microglia	HSV-1	• Upregulated AD-related genes associated with Aβ clearance, RNA metabolism, and mitochondrial function	Sundstrom et al. (2024)
BBB + neurovascular unit-on-a-chip	• Static culture • Periodic medium refreshment • Hydrogel-embedded (Matrigel)	• Human brain microvascular endothelial, neuroblastoma, astrocyte, and microglia cell lines • Primary peripheral blood mononuclear cells	HSV-1	• BBB penetration and increased permeability • BBB transmigration of blood mononuclear cells • Aβ42 accumulation • Neuron/astrocyte infection, pro-inflammatory activation, apoptosis • Microglial activation and enhanced phagocytosis	Zhang et al. (2025)
Cortical organoid	• Dynamic culture (orbital shaker) • Periodic medium refreshment • Self-aggregating	• hiPSC-derived neurons, astrocytes	SARS-CoV-2	• Astrocytes predominantly infected • Astrocytic pro-inflammatory activation • Upregulation of cell survival pathways in infected and bystander astrocytes	Andrews et al. (2022)
Cortical organoid	• Dynamic culture (bioreactors) • Periodic medium refreshment • Self-aggregating	• hiPSC-derived neurons, astrocytes	SARS-CoV-2	• Astrocytes predominantly infected • Astrocytic pro-inflammatory activation • Upregulation of cell survival pathways in infected and bystander astrocytes	Colinet et al. (2025)
Cerebral organoid	• Dynamic culture (orbital shaker) • Periodic medium refreshment • Hydrogel-embedded (Matrigel)	• hiPSC-derived neurons, astrocytes, microglia, NPCs	SARS-CoV-2	• Microglia were exclusively infected by the original, delta, and omicron SARS-CoV-2 strains	Kase et al. (2023)
Cerebral organoid	• Dynamic culture (orbital shaker) • Periodic medium refreshment • Hydrogel-embedded (Matrigel)	• hiPSC-derived neurons, NPCs, radial glia • Primary human astrocytes	SARS-CoV-2	• Dysfunction and loss of uninfected bystander neurons • Neuropilin-1-mediated cell entry of SARS-CoV-2 • Astrocytes predominantly infected • Astrogliosis and pro-inflammatory activation	Kong et al. (2022)
Assembloid (cortical + blood vessel organoids)	• Dynamic culture (orbital shaker) • Periodic medium refreshment • Self-aggregation and fusion	• hiPSC-derived neurons, astrocytes, microglia, pericytes, endothelial cells, vascular smooth muscle cells	SARS-CoV-2	• Aβ plaque formation • Neuronal loss • Increased tau phosphorylation and mislocalization • Glial pro-inflammatory activation	Kong et al. (2023)
Cortical organoid	• Static culture • Periodic medium refreshment • Self-aggregating	• hESC-derived neurons, astrocytes, radial glia progenitors	SARS-CoV-2	• Glia predominantly targeted • Infected cells upregulated apoptotic markers	McMahon et al. (2021)
Brain organoid	• Dynamic culture (spinner flasks) • Periodic medium refreshment • Hydrogel-embedded (Matrigel)	• hiPSC-derived neurons, astrocytes, microglia	SARS-CoV-2	• Neuronal infection and loss • Tau hyperphosphorylation and axon-to-soma translocation	Ramani et al. (2020)
Brain organoid	• Static and dynamic culture (orbital shaker and bioreactor) • Periodic medium refreshment • Hydrogel-embedded (Matrigel)	• hiPSC-derived neurons, astrocytes, microglia, NPCs, neural crest cells	SARS-CoV-2	• Neuronal cell death • Microglial pro-inflammatory activation and threefold increase in synaptophagy	Samudyata et al. (2022)
BBB-on-a-chip	• Dynamic culture (microfluidics) • Continuous medium refreshment • Hydrogel-embedded (collagen, hyaluronan, Matrigel)	• Primary human brain microvascular endothelial cells	SARS-CoV-2 (S1 and S2 subunits)	• Increased BBB permeability • Pro-inflammatory activation • Upregulation of matrix metalloproteinases	Buzhdygan et al. (2020)
BBB-on-a-chip	• Dynamic culture (microfluidics) • Continuous medium refreshment • Hydrogel-embedded (collagen, hyaluronan, Matrigel)	• Human brain microvascular endothelial cell line	SARS-CoV-2 (S1 subunit)	• Impaired BBB homeostasis and permeability mediated by RhoA activation and ACE2 expression	DeOre et al. (2021)
BBB-on-a-chip	• Static culture • Periodic medium refreshment • Membrane-supported	• Human cerebral microvascular endothelial, astrocyte, brain vascular pericyte cell lines	SARS-CoV-2 envelope (S2E) protein	• BBB penetration and impaired permeability • Decreased cellular viability • Astrocytic and endothelial cell pro-inflammatory response	Ju et al. (2022)
BBB + neurovascular unit-on-a-chip	• Dynamic culture (microfluidics) • Continuous medium refreshment • Hydrogel-embedded (collagen)	• Human pericyte and astrocyte cell lines • Primary brain-derived microvascular endothelial cells • hiPSC-derived neurons	TNF-α LPS IL-1β	• Increased BBB permeability • Reduction and diffusion of BBB tight junctions • Increased cytokine production • Altered metabolic signature profiles	Brown et al. (2016)
BBB-on-a-chip	• Dynamic culture (microfluidics) • Continuous medium refreshment • Membrane-supported	• Human brain microvascular endothelial cell line	TNF-α	• Decreased transendothelial electrical resistance	Griep et al. (2013)
BBB + neurovascular unit-on-a-chip	• Dynamic culture (microfluidics) • Continuous medium refreshment • Membrane-supported	• hiPSC-derived neurons, brain microvascular endothelial-like cells • Primary human astrocytes, brain pericytes • Human microglia cell line	TNF-α	• Increased BBB permeability • Glial and pericyte pro-inflammatory activation	Pediaditakis et al. (2022)
BBB + neurovascular unit-on-a-chip	• Dynamic culture (microfluidics) • Continuous medium refreshment • Membrane-supported	• hiPSC-derived neurons, astrocytes, brain microvascular endothelial-like cells • Primary human brain astrocytes, vascular pericytes	TNF-α IL-1β IL-8	• Increased BBB permeability • Altered expression of the tight junction marker ZO-1 • Retraction of astrocytic protrusions and reduced vascular endfeet coverage	Vatine et al. (2019)
BrainSphere	• Dynamic culture (orbital shaker) • Periodic medium refreshment • Self-aggregating	• hiPSC-derived neurons, astrocytes, oligodendrocytes • Human microglia cell line	Zika virus Dengue virus Flavivirus	• Microglial pro-inflammatory activation	Abreu et al. (2018)
Cerebral organoid	• Dynamic culture (orbital shaker) • Periodic medium refreshment • Hydrogel-embedded (Matrigel)	• AD patient iPSC-derived neurons, astrocytes	Zika virus	• Increased neuronal apoptosis, Aβ production, tau phosphorylation, endoplasmic reticulum stress	Lee et al. (2022)
Cerebral organoid	• Static culture • Periodic medium refreshment • Self-aggregating	• hiPSC-derived neurons, astrocytes, microglia	Zika virus	• Microglial pro-inflammatory activation and excessive synaptophagy • Astrogliosis	Xu et al. (2021)
(2) Environmental toxicants					
Cerebral organoid	• Dynamic culture (orbital shaker) • Periodic medium refreshment • Self-aggregating	• hESC-derived neurons, astrocytes, NPCs	Cadmium	• Astrocytic pro-inflammatory activation	Huang et al. (2021)
Cerebral organoid	• Dynamic culture (orbital shaker) • Periodic medium refreshment • Hydrogel-embedded (Matrigel)	• hiPSC-derived neurons, NPCs	DPM	• Neuronal network dysfunction • Reduced pre– and post-synaptic proteins • Neurotransmitter imbalance	Park and Choi (2023)
Brain-on-a-chip	• Static culture • Periodic medium refreshment • Hydrogel-embedded (Matrigel)	• Human NPC-derived neurons, astrocytes • Human microglia cell line	DPM	• Astrogliosis • Microglial migration and pro-inflammatory activation, leading to synaptic damage, accumulation of phosphorylated tau, and neuronal loss	Kang et al. (2021)
Brain-on-a-chip	• Dynamic culture (microfluidics) • Continuous medium refreshment • Hydrogel-embedded (collagen, laminin, Matrigel)	• Human NPC-derived neurons, astrocytes • Human microglial and brain microvascular endothelial cell lines	DPM	• Aβ accumulation and tau hyperphosphorylation • Neuronal hyperactivity and reduced viability • Astrogliosis • Microglial pro-inflammatory activation and overproduction of H2O2/ROS • Vascular disruption and increased permeability	Seo et al. (2023)
BBB-on-a-chip	• Dynamic culture (microfluidics) • Periodic medium refreshment • Hydrogel-embedded (fibrin)	• Human vascular umbilical endothelial cells, astrocytes	Indoor nanoscale PM	• BBB penetration • Astrocytic activation, gliosis and ROS overproduction • Reduced astrocytic viability	Li et al. (2019)
Cerebral organoid	• Dynamic culture (orbital shaker) • Periodic medium refreshment • Self-aggregating	• hiPSC-derived neurons, astrocytes, microglia	Microplastics	• Organoid penetration • Elevated apoptotic response • Upregulation of neurotoxicity-related genes • Altered expression of metabolism-related genes	Park et al. (2025)
Cerebral organoid	• Dynamic culture (orbital shaker) • Periodic medium refreshment • Self-aggregating	• hESC-derived neurons, astrocytes, radial glia, NPCs	PFAS	• Neuronal network dysfunction, apoptosis • Aβ accumulation • Tau hyperphosphorylation • Disrupted lipid metabolism	Lu et al. (2024)
(3) Gut-brain axis and microbiota-related factors					
Cerebral organoid	• Dynamic culture (orbital shaker) • Periodic medium refreshment • Hydrogel-embedded (Matrigel)	• hiPSC-derived neurons, astrocytes, NPCs	Pathogenic microbiota	• Organoid structural disruption • Neuronal loss, impaired energy metabolism, increased production of AD-related proteins	Isik et al. (2025)
GBA-on-a-chip	• Dynamic culture (microfluidics) • Continuous medium refreshment • Membrane-supported	• hiPSC-derived neurons, NPCs • Human intestinal epithelial cell line	Probiotic gut microbe-derived metabolites and exosomes	• Promotion of neuronal differentiation, maturation, synaptogenesis and plasticity • Mitigation of Aβ-induced effects on axon growth and synaptic plasticity	Kim et al. (2024)

Abbreviations: Aβ, amyloid beta; AD, Alzheimer’s disease; BBB, blood-brain barrier; DPM, diesel particulate matter; GBA, gut-brain axis; hESC, human embryonic stem cell; hiPSC, human induced pluripotent stem cell; HIV-1, human immunodeficiency virus type 1; hNSC, human neural stem cell; HSV-1, human simplex virus type 1; NPCs, neural progenitor cells; PEGDMA, poly(ethylene glycol) diacrylate; PFAS, Per- and polyfluoroalkyl substances; PM, particulate matter; ROS, reactive oxygen species; SARS-CoV-2, severe acute respiratory syndrome coronavirus 2; ZIKV, zika virus.

## 2 CIVMs to explore novel Alzheimer’s disease etiological hypotheses and risk factors

### 2.1 CIVMs to explore the impact of pathogen infections on neuroinflammation and AD

The infectious hypothesis posits that pathogens such as viruses, bacteria, and fungi can enter or persist within the central nervous system by crossing the blood-brain barrier (BBB) and eliciting an immune response. Growing evidence suggests that pathogenic infections may contribute to AD pathogenesis by triggering or exacerbating neuroinflammatory responses that eventually develop chronicity, with subsequent microglial dysfunction leading to synaptic loss, neuronal death, and overall cognitive decline (Seaks and Wilcock, 2020; Catumbela et al., 2023). Multiple studies suggest that Aβ itself may function as an antimicrobial peptide by forming aggregates which entrap invading pathogens (Kumar et al., 2016; Gosztyla et al., 2018; Prosswimmer et al., 2024). Amyloidogenicity could subsequently emerge as plaque accumulation outstrips the microglial capacity for clearance, which naturally declines with ageing.

Organoids comprising neurons and glia display robust astrocytic and microglial activation upon exposure to viruses such as human immunodeficiency virus type 1 (HIV-1) (Dos Reis et al., 2020; Boreland et al., 2024; Kong et al., 2024; Martinez-Meza et al., 2025; Narasipura et al., 2025), human simplex virus type 1 (HSV-1) (Cairns et al., 2020; Qiao et al., 2020; Qiao et al., 2022; Sundstrom et al., 2024), severe acute respiratory syndrome coronavirus 2 (SARS-CoV-2) (Andrews et al., 2022; Kong et al., 2022; Kong et al., 2023; Colinet et al., 2025), and Zika virus (ZIKV) (Abreu et al., 2018; Xu et al., 2021), leading to the upregulation of innate immune signaling pathways and increased secretion of pro-inflammatory cytokines and chemokines.

Viral infection can also replicate key neuropathological hallmarks of AD in 3D human brain organoids, including increased Aβ accumulation and tau phosphorylation, as seen in HSV-1 (Abrahamson et al., 2021; Ijezie et al., 2024; Cairns et al., 2025; Oh et al., 2025) and ZIKV (Lee et al., 2022) treatments. Additionally, Aβ oligomers bind to HSV-1 surface glycoproteins, leading to increased Aβ production and HSV-1 entrapment (Eimer et al., 2018). In SARS-CoV-2, glia are the predominantly infected cell type in human brain organoids (McMahon et al., 2021; Kase et al., 2023), with microglia-mediated synaptic engulfment increasing by threefold (Samudyata et al., 2022). Neuronal tau hyperphosphorylation and translocation to the soma is also observed (Ramani et al., 2020), highlighting how organoid models can be leveraged to disentangle the cellular and molecular mechanisms of viral infection in the human brain and its potential role in AD onset.

Microfluidic systems have become effective for modeling the BBB under physiologically relevant conditions by recapitulating key biomechanical properties such as flow rate, fluidic shear stress, and the formation of endothelial tight junctions (Shin et al., 2019). Due to their capacity for triggering neuroinflammatory responses, lipopolysaccharide (LPS) and tumor necrosis factor-alpha (TNF-α) are commonly used stimuli to mimic aspects of infection *in vitro*. In brain-on-a-chip models of the BBB, both have been associated with increased permeabilization. LPS treatment reduces the prevalence of tight junctions (Brown et al., 2016), whereas TNF-α alters the expression of endothelial tight junction markers (Vatine et al., 2019), decreases transendothelial electrical resistance by around tenfold (Griep et al., 2013), and increases cytokine production, all of which contribute to BBB leakage (Pediaditakis et al., 2022). Importantly, BBB damage and impaired cerebral blood flow have been described as early pathological hallmarks of neurodegeneration leading to AD (Nortley et al., 2019; Sousa et al., 2023).

HSV-1 infection of co-cultured human microvascular endothelial cells, astrocytes, microglia, and neurons within a multi-compartment chip induced increased BBB permeability, pro-inflammatory cytokine production, and neuron-glia apoptosis (Zhang et al., 2025). Similarly, SARS-CoV-2 infection perturbs BBB homeostasis, reducing the viability of neurovascular cells and eliciting prolonged pro-inflammatory responses (Buzhdygan et al., 2020; DeOre et al., 2021; Ju et al., 2022). Human brain-on-a-chip systems are also suitable for evaluating antiviral therapeutics (Shin et al., 2019; Boghdeh et al., 2022), some of which may hold potential for AD treatment (Iqbal et al., 2020; Drinkall et al., 2025).

### 2.2 CIVMs to explore the impact of environmental pollutants on neurodegeneration and AD

Environmental toxicants such as air, water, and soil pollutants are globally pervasive, with chronic human exposure posing considerable risks to long-term neurological health (Nabi and Tabassum, 2022). Evidence from both *in vitro* and *in vivo* studies has demonstrated their ability to disrupt neural cell homeostasis (Iqubal et al., 2020). The accumulation of hyperphosphorylated tau and Aβ is also observed across multiple studies, suggesting that chronic toxicant-induced neuropathology may contribute to the development of AD (Dhapola et al., 2024). Given the complexity and underlying interrelatedness of these adverse cellular events, CIVMs offer a human-relevant platform for mechanistically unraveling how environmental toxicants contribute to neurodegenerative processes. Examples of studies examining the neurotoxic and neurodegenerative effects of some well-known environmental pollutants in neuronal and glial CIVMs are reported in this section.

Cadmium, a common heavy metal pollutant in industrial emissions and phosphate fertilizers, induces an acute neuroinflammatory response in human embryonic stem cell (hESC)-derived brain organoids, evidenced by widespread glial activation and increased IL-6 production (Huang et al., 2021).

Diesel particulate matter (DPM) is a major ambient air pollutant produced by combustion engines, with hiPSC-derived brain organoid exposure resulting in altered neuronal electrophysiological signaling, synaptic damage, and increases in inflammatory markers (Park and Choi, 2023), all of which have been associated with AD (Nordengen et al., 2019; Meftah and Gan, 2023).

Per- and polyfluoroalkyl substances (PFAS), also known as ‘forever chemicals’, consist of over 7 million synthetic organofluorine compounds that are widely used to enhance the water-, grease-, and heat-resistance of commercial and industrial products. They have become pervasive in global water sources, with some retaining a half-life of over 8 years within the human body (Buck et al., 2011). Chronic PFAS exposure in hESC-derived cerebral organoids over 35–70 days increased both tau phosphorylation and Aβ accumulation (Lu et al., 2024).

Lastly, the ubiquity of microplastics in terrestrial and aquatic ecosystems has become a topic of global interest, raising questions about their potential neurotoxicity. Exposure of hiPSC-derived brain organoids to microplastic beads (50–100 μm) over 3 weeks significantly reduced cellular viability and cholinergic-related acetylcholine levels, indicating disrupted neuronal signaling and potential synaptic dysfunction (Park et al., 2025).

Environmental toxicants can also be incorporated into microfluidic “brain-on-a-chip” systems to investigate their neuropathological mechanisms. In models comprising human brain endothelial cells, neurons, and glia, DPM exposure induced tau hyperphosphorylation, Aβ accumulation, neuronal cell death and astrogliosis, along with microglial activation and overproduction of reactive oxygen species (ROS) (Kang et al., 2021; Seo et al., 2023).

With people in developed countries typically spending 80%–90% of their time indoors, indoor airborne particulate matter (PM) also constitutes an important source of chronic pollutant exposure (Duffield and Bunn, 2023). Li et al. (2019) treated a human BBB-on-a-chip model comprising astrocytes and endothelial cells with indoor nanoscale PM retrieved from non-smoking residences in Wuhan, China. They demonstrated that indoor nanoscale PM traversed the endothelial barrier before inducing abnormal astrocytic proliferation and elevated ROS production, while also reducing overall cellular viability. This highlights the alarming effects that ambient indoor PM exposure can have on the development of chronic neuroinflammation.

### 2.3 CIVMs to explore the impact of microbiota and gut-brain axis alteration on AD onset

The GBA is a bidirectional communication network that links the central nervous system with the enteric nervous system, gastrointestinal tract, and gut microbiota. Signaling occurs via metabolic, immune, and neural pathways, with homeostasis across the GBA underpinning normal physiological function (Cryan et al., 2019). GBA disruption can arise through microbial dysbiosis, such as increases in pro-inflammatory bacterial taxa (Ashique et al., 2024), and has been increasingly linked to neurodegenerative processes including neuroinflammation, Aβ deposition, and tau pathology (Wang et al., 2014; Erny et al., 2015; Seo and Holtzman, 2024).

The implementation of 3D human brain organoids to model the GBA remains in its infancy. One promising strategy involves using transwell systems, which allow for vesicle trafficking and molecular diffusion across a semi-permeable membrane (Alam et al., 2024). The co-culture of hiPSC-derived brain organoids and pooled pathogenic microbiota led to reduced neuronal viability, upregulation of AD-associated genes, and disruption of the organoid’s structural integrity (Isik et al., 2025), reflecting how gut dysbiosis may promote neurodegeneration. Other proposed organoid-based GBA models include the direct exposure of cerebral organoids to gut microbiota-conditioned medium, or co-culturing cerebral and intestinal organoids within transwell systems separated by an endothelial cell layer to mimic the BBB (Alam et al., 2024).

Single- and multi-OOC microfluidic systems are increasingly being applied to model the GBA, with the European Research Council-funded MINERVA project (grant agreement ID: 724734) representing a major milestone. The project integrated five interconnected hiPSC-derived OOC modules (microbiota, gut epithelium, immune system, BBB, and brain) to recapitulate communication along the GBA (Raimondi et al., 2019). Other microfluidic models using human-derived cells have also been established to study exosomal transport across the BBB (Kim et al., 2021; Seo et al., 2024). Notably, exosomes and metabolites derived from probiotic *Lactobacillus casei* and *L. plantarum* bacteria were shown to promote synaptic plasticity, suggesting the therapeutic potential of microbiota-derived factors in mitigating neurodegenerative processes (Kim et al., 2024). Although direct applications to AD-related mechanisms have been limited, these microfluidic GBA platforms have laid the foundations for investigating microbiota-mediated mechanisms of neuroinflammation and amyloidogenicity under controlled and human-relevant conditions (Kandpal et al., 2024).

## 3 Discussion

Despite decades of research investment, the number of drugs available for managing AD remains limited, with most providing only symptomatic relief and benefiting a restricted subset of patients (Oxford et al., 2020). The recent approval of amyloid-targeting antibodies marks an important step toward disease-modifying therapies. However, these treatments are indicated primarily for individuals with early-stage AD or mild cognitive impairment, and they are not curative, carrying their own risks and limitations^5^.

New hypotheses have emerged to explain the complex etiopathology of AD (Zhang et al., 2024), which should be taken into account when designing novel therapeutic and preventive strategies. This is particularly relevant given that nearly half of all dementia cases could be prevented by addressing modifiable risk factors (Livingston et al., 2024).

The high historical failure rate in AD drug development (Cummings et al., 2014) may have stemmed from an overreliance on reductionist disease hypotheses and inadequate preclinical models, including transgenic animals and simplistic *in vitro* systems. Emerging human-centered models now offer powerful tools to elucidate the role of risk factors in triggering and exacerbating neurodegeneration and AD (Sreenivasamurthy et al., 2023; Lopes and Guil-Guerrero, 2025; Dolciotti et al., 2025). Particularly in the field of AD research, where animal experimentation continues to feature prominently, CIVMs have been at the forefront of a recent paradigm shift towards the broader adoption of human-centered and non-animal methodologies (Taylor et al., 2024; Vashishat et al., 2024) to drive progress and increase the translatability of preclinical research findings (Mehta et al., 2025).

The enhanced applicability of CIVMs for modeling human biology at both cellular and molecular levels has progressively enabled comprehensive investigations into novel hypotheses underlying AD etiology. These include neuroinflammatory responses to pathogens (Seaks and Wilcock, 2020; Catumbela et al., 2023), the effects of environmental toxicants (Dhapola et al., 2024), and the involvement of interactions along the GBA (Seo and Holtzman, 2024), which are increasingly recognized as potential drivers of neurodegeneration. As reported in this review, human-centered CIVMs, particularly cerebral organoids and OOC systems, can be applied to explore these new etiological hypotheses.

Each model has its strengths and weaknesses. CIVMs offer the potential advantage of being patient-specific, preserving individual (epi)genetic traits that support personalized and precision medicine approaches (Lopes and Guil-Guerrero, 2025). While they hold promise for overcoming the constraints of animal and simplistic *in vitro* models, technical optimization remains essential to realize their full potential. For example, brain organoids model early brain development rather than the aging brain, so their relevance to age-related neurodegeneration remains uncertain and needs rigorous validation through complementary approaches (Cerneckis et al., 2023). In addition, lack of vascularization can lead to internal hypoxia and cellular stress, resulting in necrosis and the impaired specification of cellular subtypes (Parthasarathy et al., 2026). CIVMs also face reproducibility issues due to biological variability, limited standardization and scalability, and poor reporting, hindering their validation and adoption by industry (Pamies et al., 2024).

Despite these limitations, legislation in the EU (European Medicines Agency, 2023), the US (Food and Drug Administration, 2024; Food and Drug Administration, 2025) and other regions is increasingly supporting the integration of non-animal approaches in pharmaceutical development, signaling a broader shift toward human-centered research.

Recent evidence suggests that dynamic culture conditions can substantially improve the physiological relevance of *in vitro* models. For example, perfusion-based systems have been shown to better recapitulate *in vivo* vascularization by enhancing nutrient delivery, waste removal, and biomechanical cues compared with static culture (Quintard et al., 2024). Similarly, oxygenation has been identified as a critical determinant of organoid viability and maturation, with insufficient oxygen supply markedly impairing cellular health and disrupting normal tissue development in brain organoids (Leung et al., 2022; Mohapatra et al., 2025). Moreover, variations in extrinsic forces and spatio-temporal dynamics can influence cellular composition and metabolic parameters in brain organoids (Aiello et al., 2025), thereby affecting both synaptogenesis and the proportion of specialized neuronal cells and glia (Saglam-Metiner et al., 2023), which represent critical endpoints for faithfully modeling neurodegenerative conditions and AD. Together, this highlights that microenvironmental parameters are central to maintaining homeostatic and pathophysiological processes in complex 3D cultures. Therefore, it is imperative not only to optimise CIVM culture parameters systematically, but also to implement rigorous, longitudinal characterisation of these test systems. Such characterisation should include quantitative assessment of culture conditions, cell viability, and disease-relevant biomarkers to ensure reproducibility and predictive validity.

To enhance reproducibility, fit-for-purpose guidelines have been introduced to promote the consistent performance of CIVMs (Pamies et al., 2024). Furthermore, minimum reporting standards for *in vitro* models, including organoids and microphysiological systems (Pamies et al., 2018; Mohapatra et al., 2025), have been proposed to enhance study quality for safety and regulatory assessments, with relevance also for basic and translational research and drug efficacy testing.

We acknowledge two main limitations of this study. Firstly, due to word count constraints, only studies employing organoids and OOC models were considered, whereas other CIVMs such as co-culture models could also be relevant. Secondly, for the same reason, we focused on selected hypotheses regarding AD pathogenesis, while recognizing that CIVMs have also been used to investigate additional etiological hypotheses not addressed in this review.

## 4 Conclusion and future outlook

CIVMs are playing an increasingly pivotal role in AD research. Their capacity to recapitulate key aspects of human brain physiology and pathology offers unprecedented opportunities to deepen our understanding of AD etiopathogenesis, including the contribution of genetic, molecular, and environmental risk factors.

Modern research increasingly combines CIVMs with advanced computational approaches, AI, and digital twin technologies to integrate and interpret the rapidly expanding body of biological and clinical data, thereby accelerating target discovery and drug development (Li et al., 2018; Cheng et al., 2025). Such integrative frameworks hold great promise for revealing novel disease mechanisms and optimizing therapeutic interventions in a human-relevant context.

Future research should focus on disentangling correlations from causal relationships among risk factors and comorbidities to more precisely identify pathogens and environmental contributors that directly drive neurodegeneration and AD onset. Elucidating these causal pathways at the molecular and cellular levels will be critical for developing effective preventive and therapeutic strategies. These efforts should be underpinned by the broader adoption of human-centered CIVMs and other new approach methodologies which can improve translatability, reduce reliance on animal models, and enable truly patient-centered approaches to AD research, prevention, and treatment development.

## Footnotes

1
https://www.who.int/news-room/fact-sheets/detail/dementia

2
https://www.alzint.org/about/dementia-facts-figures/dementia-statistics/

3
https://www.alz.org/alzheimers-dementia/treatments/medications-for-memory

4
https://www.mayoclinic.org/diseases-conditions/alzheimers-disease/in-depth/alzheimers/art-20048103

5
https://www.mayoclinic.org/diseases-conditions/alzheimers-disease/in-depth/alzheimers/art-20048103
